# Genome-wide RNAi screens in African trypanosomes identify the nifurtimox activator NTR and the eflornithine transporter AAT6

**DOI:** 10.1016/j.molbiopara.2010.11.010

**Published:** 2011-03

**Authors:** Nicola Baker, Sam Alsford, David Horn

**Affiliations:** London School of Hygiene & Tropical Medicine, Keppel Street, London WC1E 7HT, UK

**Keywords:** DFMO, Eflornithine, Nifurtimox, Ornidyl, Resistance, *Trypanosoma brucei*

## Abstract

To be effective, therapeutic compounds must typically enter target cells and, in some cases, must be concentrated or modified. Thus, uptake and activation mechanisms often form the basis of selectivity against infectious agents. Loss-of-function screens can be used to identify proteins involved in drug uptake and metabolism and may also identify clinically relevant potential resistance mechanisms. We used a genome-scale RNA interference (RNAi) library to identify loss-of-function resistance mechanisms in bloodstream-form *Trypanosoma brucei*. Nifurtimox–Eflornithine Combination Therapy (NECT) was recently introduced for Human African Trypanosomiasis and we focus on these drugs here. Screens for resistance to nifurtimox and a related drug, benznidazole, identified loss of nitroreductase (NTR) pro-drug activator function. A screen for resistance to the amino-acid analogue, eflornithine, identified loss of amino-acid transporter (AAT6) function. Our results confirm recent findings and suggest that NTR or AAT6 loss-of-function represent major potential mechanisms of resistance to these drugs. Thus, bloodstream-form *T. brucei* RNAi libraries present a versatile tool for selective genetic screening and for the rapid identification of drug-activation, uptake and potential resistance mechanisms.

## Graphical abstract

**Graphical abstract:**

Genome-scale RNA interference (RNAi) library screens in bloodstream-form *Trypanosoma brucei*, using nifurtimox and benznidazole, revealed type I nitroreductase, NTR, knockdown. A similar screen with eflornithine, revealed amino-acid transporter, AAT6, knockdown. .

**Research highlights:**

▶ A genome-scale RNA interference library is used to identify loss-of-function resistance mechanisms in bloodstream-form *Trypanosoma brucei*. ▶ Screens for resistance to nifurtimox or benznidazole identified loss of nitroreductase function. ▶ A screen for resistance to eflornithine identified loss of amino-acid transporter function.

Drug resistance arises when a genetic change confers a selective advantage through, for example, loss or reduction of an uptake mechanism. Provided that the genetic change does not have a major negative impact on growth, the mutant will proliferate and come to dominate in a selective environment. Any resulting spread of resistance then renders the relevant drug ineffective. There are few drugs available to treat the diseases caused by African trypanosomes and drug resistance is an increasing problem [1]. Little is known about drug uptake and metabolism, or about mechanisms of resistance, and few new drugs have been introduced in recent years. Eflornithine was introduced in the 1980s to treat late-stage disease in West and central Africa [2], and more recently has been combined with nifurtimox [3]. This Nifurtimox–Eflornithine Combination Therapy (NECT) regime is simpler to administer than eflornithine alone, and may prolong the usefulness of both drugs. Recently, the trypanocidal effects of nifurtimox and of benznidazole, a compound closely related to nifurtimox, was shown to be mediated by a nitroreductase (NTR), thought to be required to generate toxic intermediates in both African and South American trypanosomes [4]. Even more recently, eflornithine uptake was shown to be mediated by an amino acid transporter [5,6]. Using nifurtimox, benznidazole and eflornithine selection, we report a method for the rapid identification of resistance mechanisms in bloodstream-form *Trypanosoma brucei*, likely to reflect potential mechanisms of resistance in the closely related human-infective trypanosomes, *T. b. gambiense* and *T. b. rhodesiense*.

RNA interference library screens essentially combine forward and reverse genetics, constituting powerful systems for linking genes to function. Such approaches have proven invaluable in the post-genomic era. In selective RNAi screens, cells displaying loss-of-function phenotypes are enriched under selective pressure. Englund and colleagues introduced this concept in insect-stage African trypanosomes in 2002 [7]. Other systems have followed and have subsequently been widely used [8]. To investigate phenotypes in bloodstream-form *T. brucei*, we used a high-efficiency transfection technology to make RNAi libraries in this life-cycle stage that virtually eliminates position effects [9]. A separate study with the first of these libraries demonstrated good genome coverage with each gene represented by >5 independent RNAi target fragments on average [10]. Here, we report initial proof-of-principle for RNAi library screening and the identification of drug resistance mechanisms. Enriched RNAi target fragments were sequenced to reveal the candidate genes which, when knocked down, confer resistance. Since background mutation could generate false positives, only genes targeted by multiple independent RNAi fragments were considered as validated ‘hits’. This approach in three separate screens revealed genes recently linked to drug resistance.

Our 10-day protocol involved exposing the library to a brief period of RNAi induction, then a period of induction plus drug selection and, finally, recovery of enriched RNAi target fragments (Fig. 1A). As expected, exposure to nifurtimox (Fig. 1B) or eflornithine (Fig. 1C) selection resulted in a period of curtailed growth followed by outgrowth of a resistant population, first apparent after 6 days under selective pressure (Fig. 1B and C). Genomic DNA was extracted from each resistant population and the RNAi target fragments were recovered using a PCR protocol. The results revealed low-complexity products with five major products visible in the nifurtimox-selected sample (Fig. 2A) and two major products visible in the eflornithine-selected sample (Fig. 2B). All seven fragments were sequenced and mapped to the reference genome sequence [11]. A pair of nifurtimox resistance-associated fragments mapped to the type I nitroreductase protein-coding sequence, *NTR* (Fig. 2C), and both eflornithine resistance-associated fragments mapped to the amino acid transporter protein-coding sequence, *AAT6* (Fig. 2D), one of a family of 17 *AAT* genes identified in *T. brucei* [12]. A screen using benznidazole revealed a similar pattern of fragments to nifurtimox that also included the same pair of *NTR* sequences (data not shown). Additional resistance mechanisms could be revealed through further DNA sequencing or by selecting library fractions but we have not taken that approach here.

Identification of the *NTR* gene was not unexpected since this gene was previously linked to nifurtimox and benznidazole cross-resistance [4]. Since eflornithine is an amino acid analogue, it was also not surprising to identify an amino acid transporter. However, the AAT6–eflornithine association had not been described at that time and we, therefore, established independent, inducible, hairpin *AAT6* RNAi knockdown strains to confirm the association. RNAi induction increased the half-maximum effective concentration (EC_50_) for eflornithine by 16-fold (from 27 to 440 μM) in these strains (Fig. 2E). AAT6 has now been linked to eflornithine resistance by two independent groups [5,6].

Here, RNAi serves as an independent approach that reinforces and further validates the association between NTR and nifurtimox/benznidazole cross-resistance [4]. Since genome-wide RNAi screens represent an unbiased approach, identification of two independent *NTR* target fragments in both the nifurtimox and benznidazole screens strongly suggests that this enzyme is the major activator for these pro-drugs. It is notable that a drug uptake mechanism was not identified, which may reflect uptake by diffusion across membranes. These screens also indicate that genes that are essential for growth can be identified using the knockdown approach; Wilkinson and colleagues presented strong evidence that *NTR* expression was essential for the growth of bloodstream-form *T. brucei* [4]. This is an important point since clinically relevant resistance may emerge through allele loss or mutation that reduces rather than eliminates protein function. NTR depletion may also confer cross-resistance to other nitro-drugs, including fexinidazole that has recently entered clinical trials [13].

AAT6 likely plays the dominant role in eflornithine-uptake in *T. brucei* [5,6] but what are the implications for clinical resistance? Eflornithine therapy is used against West African sleeping sickness caused by *T. b. gambiense.* Uptake does not differ between *T. b. gambiense* and *T. b. rhodesiense*, but *T. b. rhodesiense* tolerates higher concentrations of eflornithine due to the shorter half-life of the target, ornithine decarboxylase [14]. The *T. brucei* and *T. b. gambiense* genome sequences [15] reveal syntenic *AAT6* genes. There is no evidence that *AAT6* loss has contributed to clinical resistance at present but both genes are flanked by almost identical calflagin coding-sequences that could facilitate *AAT6* deletion through a single-strand annealing DNA repair mechanism and *AAT6* loss was seen following *in vitro* selection for eflornithine resistance [6].

An understanding of the potential mechanisms underlying resistance and cross-resistance will be essential for the rational design of effective future therapies, will facilitate the development of approaches to surveillance and also offers insight into the basic biology of the African trypanosome. Our findings reveal potential molecular mechanisms for the emergence of clinical resistance to nitro-drugs and to eflornithine. The results also validate RNAi library screening as a means to rapidly identify drug uptake, metabolism and resistance mechanisms. Distinct mechanisms of resistance are consistent with the idea that Nifurtimox–Eflornithine Combination Therapy, rather than mono-therapy, will prolong the usefulness of both drugs.

## Figures and Tables

**Fig. 1:**
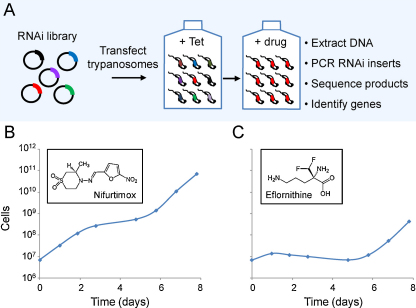
RNAi library drug resistance screens. (A) The schematic representation illustrates the RNAi library selective screening protocol. (B) Cumulative growth curve during nifurtimox selection. Bloodstream-form *T. brucei*, MiTat 1.2, clone 221a-derived libraries were maintained as described [16]; >5 × 10^6^ cells per culture at a density of <2 × 10^6^ cells ml^−1^. Tetracycline (Tet; 1 μg ml^−1^) was added at −1 day to induce RNAi, and nifurtimox (Bayer, 3 μM; 1 × EC_50_) was added at time 0. Cultures were maintained thereafter in medium containing Tet and selective drug. Genomic DNA was recovered on day 8 and increased resistance was confirmed to be Tet-dependent (data not shown). The inset shows the drug structure. (C) Cumulative growth curve during eflornithine selection. Eflornithine (Sanofi-Aventis) was applied at 70 μM (2.5 × EC_50_). Other details as in B.

**Fig. 2:**
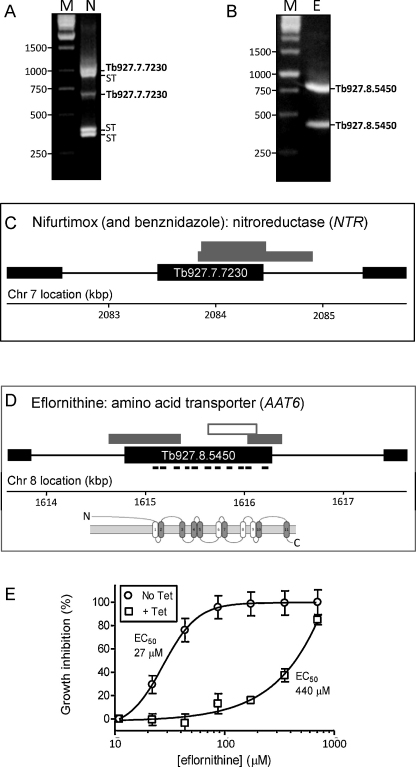
Nifurtimox and eflornithine resistance mechanisms. (A) Amplified products from the nifurtimox (N) screen. PCR amplification was carried out over 30 cycles using the primers, LIB2f (TAGCCCCTCGAGGGCCAGT) and LIB2r (GGAATTCGATATCAAGCTTGGC); 95 °C for 30 s, 57 °C for 30 s and 72 °C for 130 s. The products were separated in 1% agarose gels and then sequenced directly and also cloned to derive sequence from both ends. This confirmed that each product did indeed represent an RNAi vector-derived target fragment and also allowed precise mapping to the reference genome. M, molecular weight markers; ST, sequences that mapped to repetitive, sub-telomeric domains. (B) Amplified products from the eflornithine (E) screen. Other details as in A. (C) Genetic map (black boxes represent protein-coding sequences) indicating the location of the RNAi target fragments recovered from the library following the nifurtimox and benznidazole screens (grey boxes). (D) Genetic map indicating the location of the RNAi target fragments recovered from the library following the eflornithine screen. The RNAi target fragment used in E is represented by an open box. The locations of predicted AAT6 transmembrane-coding sequences are indicated, as well as a schematic showing the predicted transmembrane (TM) topology (open TM regions are putative). (E) AAT6 knockdown confers resistance to eflornithine. The RNAit program [17] was used to design primers and the pRPa^iSL^ construct [16] was modified to target AAT6 for RNAi. The resulting hairpin RNAi constructs were transferred to 2T1 cells [16]. For EC_50_ determination, cells were seeded at 2 × 10^3^ ml^−1^ in 96-well plates in an eflornithine 2-fold dilution series. After 66 h growth, 20 μl of Alamar blue (AbD serotec) was added to each well and the plates incubated for a further 6 h. Fluorescence was determined using a Gemini Fluorescent Plate reader (Molecular Devices) at an excitation wavelength of 530 nm, an emission wavelength of 585 nm and a filter cut-off of 570 nm [18]. Error bars indicate sd from triplicate assays. These results were confirmed using three additional independent clones (data not shown).
